# Understanding Community Participation in Rural Health Care: A Participatory Learning and Action Approach

**DOI:** 10.3389/fpubh.2022.860792

**Published:** 2022-06-06

**Authors:** Abhisek Mishra, Arvind Kumar Singh, Swayam Pragyan Parida, Somen Kumar Pradhan, Jyolsna Nair

**Affiliations:** Department of Community Medicine and Family Medicine, All India Institute of Medical Sciences, Bhubaneswar, India

**Keywords:** community participation, participatory learning and action, rural health, health system, community engagement

## Abstract

Community participation is one of the founding pillars of primary health care. However, due to various reasons, we are yet to achieve complete integration of this component into the health system functioning in India. The objective of our study was to do a formative assessment of community participation in a rural healthcare setting by adopting participatory learning action (PLA). technique. The study participants included frontline health workers and members from local governing institutions of rural areas. The study design is qualitative in nature with a participatory approach. A number of three PLA techniques have been used as a part of this study to recognize available resources for community participation, address its barriers and facilitators, and finally devise a time-line-based action plan. Based on the this, a conceptual framework for community participation pertaining to the rural healthcare system has been developed. This study highlights the importance of understanding the psychosocial aspects of community participation among various stakeholders involved in rural health care. Lessons learned from this PLA study will be helpful in the integration of community-based participatory approach within grassroot level healthcare planning and service delivery.

## Introduction

Community participation is a key factor that enables effective health system functioning and is the first step toward true community empowerment in health (1). This is one of the founding principles of Primary Health Care since Alma Ata declaration,1978. This principle reflects the underlying value of social justice in improving health, especially in deprived areas, through the involvement of the community in planning and implementation of activities toward building a healthy society (2). The way public health interventions are executed through community participation is critical to achieving a sustained improvement in health outcomes within the community (3). India is a multi-cultural, multi-ethnic developing country with diversity in health issues and challenges, which requires a sustained community participatory approach toward health compared to other countries in the world. Community participation in health has been developed and evolved in the past few decades and is rightly being augmented by the health system at the policy level in India (4, 5). Institutionalized mechanisms such as Village Health Sanitation and nutrition committees (VHNSCs), Rogi Kalyan Samiti (RKS), Panchayati Raj institutions (PRIs), Gaon Kalyan Samiti (GKS), and Self-help groups (SHGs), and so on have been the significant contributors for promoting community participation in health, especially in rural areas (6, 7). However, India is still struggling to achieve inclusive and sustained community engagement within health system due to uneven functionality and effectiveness of community participation across the country (8–10).

Planning is now considered as a “people-driven” activity, in which each level of governance has been given some degrees of self-governance and has to coordinate with each of their successive levels to implement their share of activity. This is brought the concept of micro-planning through people's participation. The “base upwards” all-inclusive planning based on the micro-planning exercises comprising multiple stakeholders could be the key toward self-resilience and demand generation (11). Incorporation of these concepts into the research ecosystem has led to the emergence of participatory methods in community-based research.

Participatory learning action (PLA) is one of these approaches which is applauded for building local capacity to work toward a healthier community. Initially, it was known as participatory rural appraisal (PRA). due to its prominent usage in rural appraisal and resource management. But later, it was adapted for other contexts such as health care, poverty, malnutrition, governance, livelihood, and so on and formally termed as “Participatory Learning Action” (PLA) (12, 13). PLA's main goal is to help people in communities analyze their situations rather than relying on outsiders to do so and to ensure that any learning is then translated into action. This strategy aids the primary stakeholders, who are often poor or from rural populations, in taking ownership throughout the process. It also allows the residents to discuss local-level issues, identify, prioritize issues or challenges, and develop practical action plans to address these complex situations. The use of PLA tools as a part of formative community engagement is a sustainable, low-tech, and labor-intensive technique for establishing ties between health workers and community people prior to the implementation of initiatives. The PLA has been contributing significantly to qualitative public health research, especially in the field of maternal and child health as well as nutrition (14, 15). Government of India has also recognized the role of PLA in empowering local institutions for identifying and planning for health-related problems within the community (16, 17).

There is a need for meaningful research, especially through a participatory approach to explore factors affecting community participation and achieving true community empowerment within the public healthcare delivery system. Many findings imply that applying PLA methodologies to the sensitive and understudied subject of gap identification and prioritization might yield contextual data on need assessment, mobilization, and group building experiences. Finally, participatory techniques can aid in the design of evaluations as well as the discovery of causal pathways and potential mechanisms for program change.

### Research Context

Odisha, a state from eastern India, is home to more than 40% socially vulnerable population with majority of them deprived in terms of key human development matrices (18). While engaging with the community in one of the rural field practice areas of All India Institute of Medical Sciences (AIIMS), Bhubaneswar, Odisha, it was observed that there is a sense of dormancy toward community participation within the population and villagers perceived health care as the responsibility of government and frontline health workers. Community participation had been limited only to Information Education and Counseling activities, thereby limiting the creative potential of community members in planning and implementation of public health interventions at the local level. This perception has been backed by evidence from various studies in India (19–21). To further enhance the understanding in this context, this study was planned through a PLA approach while involving various stakeholders associated with rural healthcare delivery at the village level. The objective of the study was to identify and explore the contextual factors embedded in the current healthcare delivery process through community participation at the village level.

## Methodology

### Study Setting

The study was conducted in rural health training center, Tangi operated under the Department of Community Medicine and Family medicine, AIIMS Bhubaneswar. A total of 10 villages were selected among 143 villages from the Tangi block of Khordha district, Odisha. The criteria used for their selection were based on average infant mortality rate during the last 5 years. It was assumed that the infant mortality rate represents the challenging health scenario of these villages (22).

### Study Design

The study design is qualitative type where PLA methods are being applied among a conveniently sampled group of participants (23). A qualitative design also provided deeper understanding of how PLA can empower the stakeholders involved in rural healthcare delivery system toward decision-making through community centered group activities.

### Study Participants

A team of resident doctors and medical social workers visited all the selected 10 villages and held discussions with key community members (PRI and GKS members, Ex-PRI and Ex-GKS members) as well as healthcare workers from those villages. At the end, 20 participants were nominated through an agreement between our team and key community members from each village. The aim of this activity in this study was to recruit individuals who were decision-makers or opinion formers or service providers from each village. The distribution and key characteristics of study participants have been explained in Table 1.

**Table 1:** Characteristics of study participants (*n*=20).

**S. No**.	**Participant**	**Frequency (%)**
**(a). Gender**		
1.	Male	5 (25)
2.	Female	15 (75)
**(b). Age group**		
1.	<30 years	5 (25)
2.	30–60 years	13 (65)
3.	>60 years	2 (10)
**(c). Educational status**		
1.	Primary to Secondary level	6 (30)
2.	Higher secondary to Intermediate level	10 (50)
3.	Graduate and above	4 (40)
**(d). Designation**		
1.	Accredited Social Health Activist (ASHA)^*^	10 (50)
2.	Gaon Kalyan Samiti (GKS). member	4 (20)
3.	Panchayati Raj Institution (PRI). member	2 (10)
4.	Self Help Group (SHG). member	4 (20)

* *Each team was having one ASHA*.

A total of four types of participants were recruited based on their designation and representation in various aspects of rural health care. The ASHA represents the implementation aspect, the GKS and PRI members project the decision-making and resource utilization aspect, and finally, the SHG member represents the community engagement aspect. We have maintained homogeneity within each group as far as the educational level of participants is concerned, which balances reflexivity in qualitative methods.

### Participatory Learning Action (PLA) Tools

We used three PLA tools for this study to understand the participant groups perception toward resource identification, planning, and implementation of solutions for health-related concerns through community participation. The activities were aimed to obtain knowledge of the situation—specifically, community stakeholders' experiences—in terms of perceived obstacles and enablers in the village, the status of various community resources, and preferences for aligning with a village health plans through community participation.

The PLA activity was spanned over 2 days with the first day being used for orientation of participants regarding the exercises followed by the PLA session and presentation on the second day. A total of 10 teams from 10 villages participated in the PLA session with three exercises for each team separately. The overall process has been summarized as Facilitation and Dissemination.

The three PLA tools used were as follows: H diagram, resource mapping, and now, sooner, and later (NSL). chart. All of them had been pilot tested in the community before their application in this exercise. The pilot testing was done in one village. They are explained in Table 2. A PLA tool guide was prepared based on validated standard methodology (24, 25).

**Table 2:** Summary and objectives of PLA techniques.

**S. No**.	**PLA tool**	**Objective and implementation**
1.	H-Diagram	The objective of this activity was to identify enablers and barriers toward community participation in health within their villages. Each team was instructed to list out facilitators in one arm and barriers in another arm of H-diagram in a chart paper.
2.	Resource mapping	The objective of this exercise was to identify the location and distances of available resources in the community contributing to health in their villages. Each team was instructed to plot the identified resources on village map in a chart paper based on the geographical direction and distance. They listed distance in terms of time duration (hours, minutes). and space (meters, kilometers).
3.	NSL (Now, Sooner, Later). chart	The objective was to classify the local solutions through community participation based on factors and resources identified in previous two activities. During this urgency and importance of the problems were also taken into consideration. All the teams were asked to prepare an action plan and put the solutions in three boxes, i.e., now (within 3 months), sooner (3 months to 1 year), and later (1 to 2 years)

#### Facilitation

**Figure 1:**
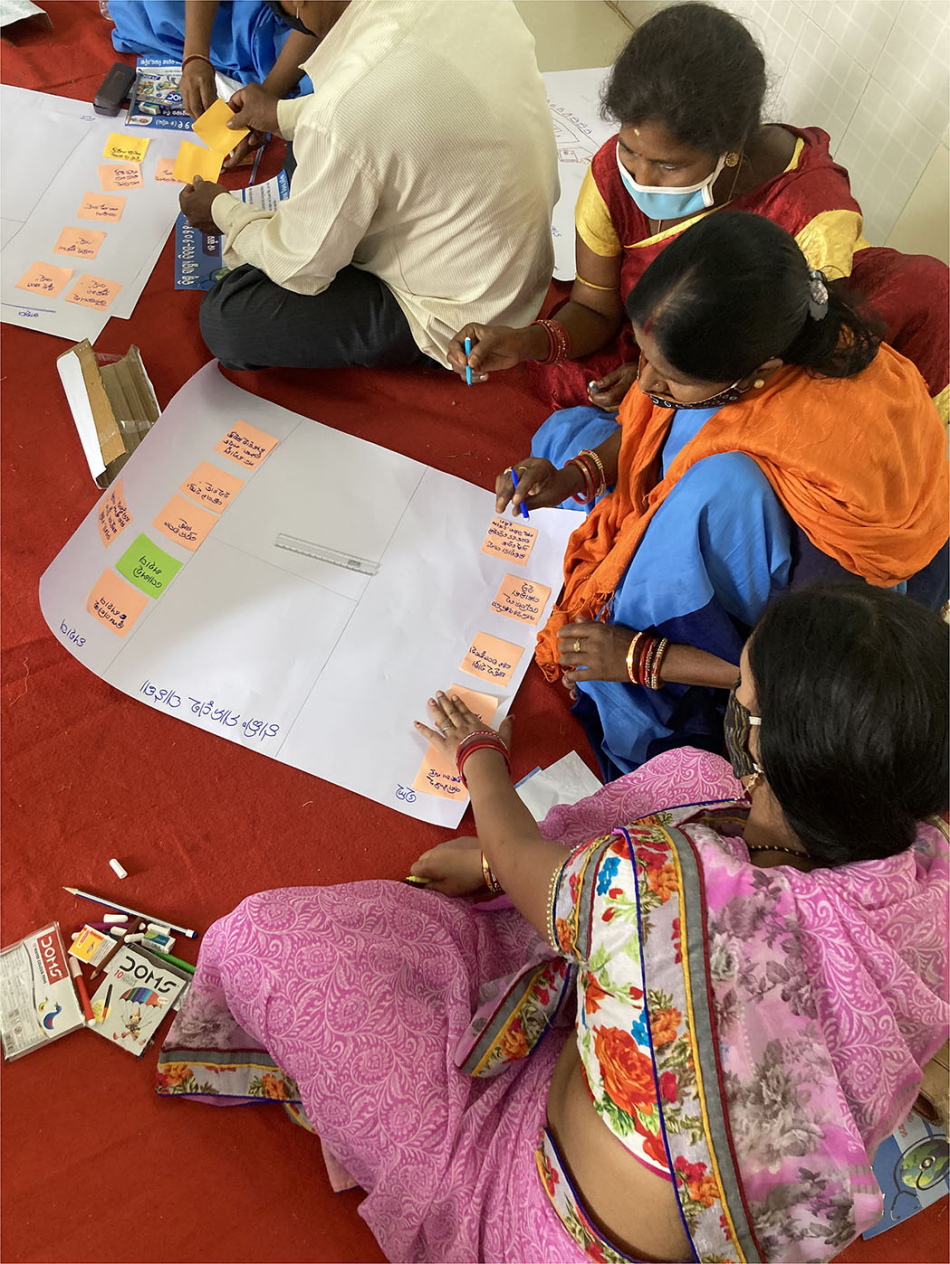
PLA activity.

**Figure 2:**
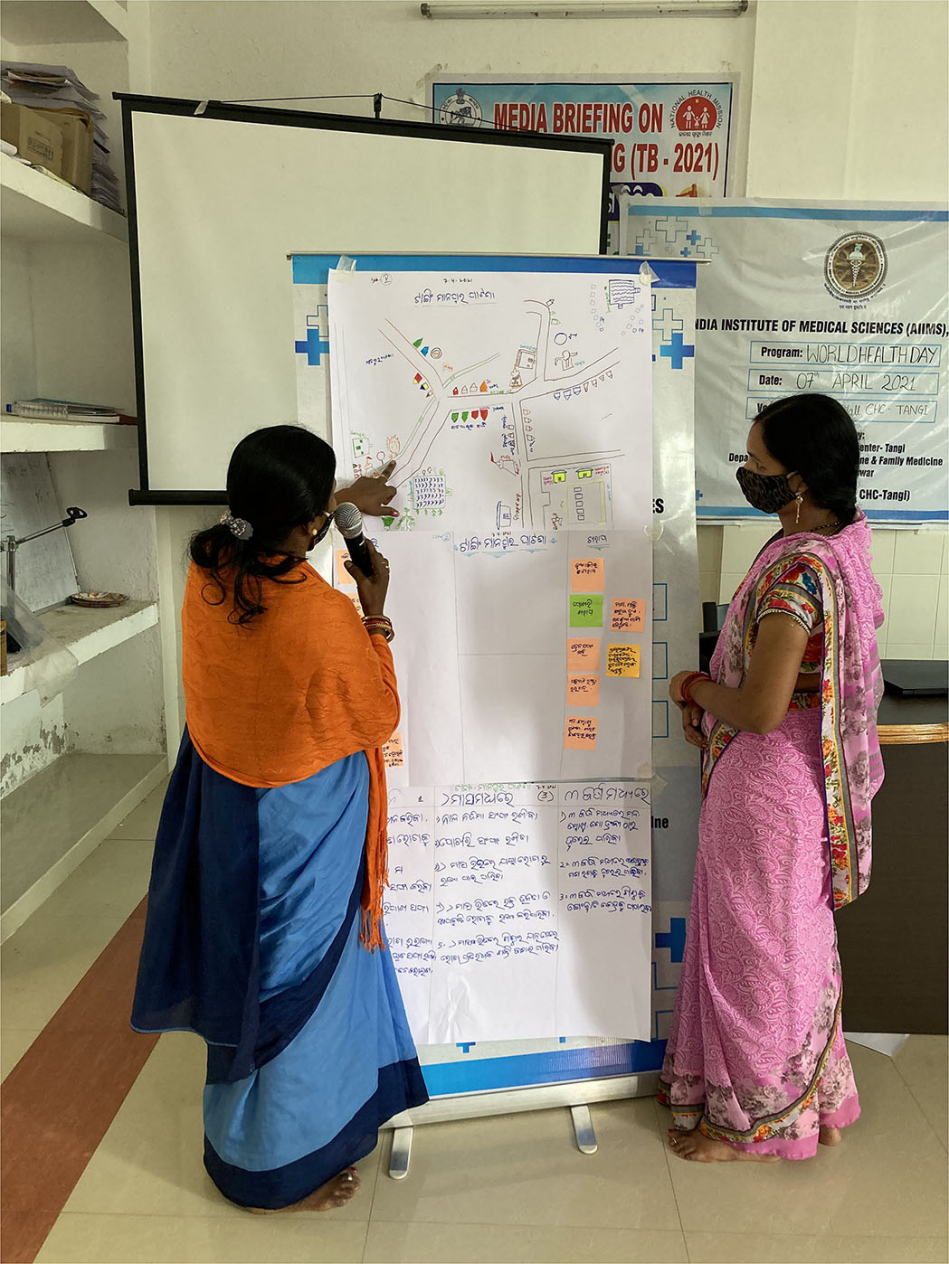
Dissemination and participant driven discussion.

**Figure 3:**
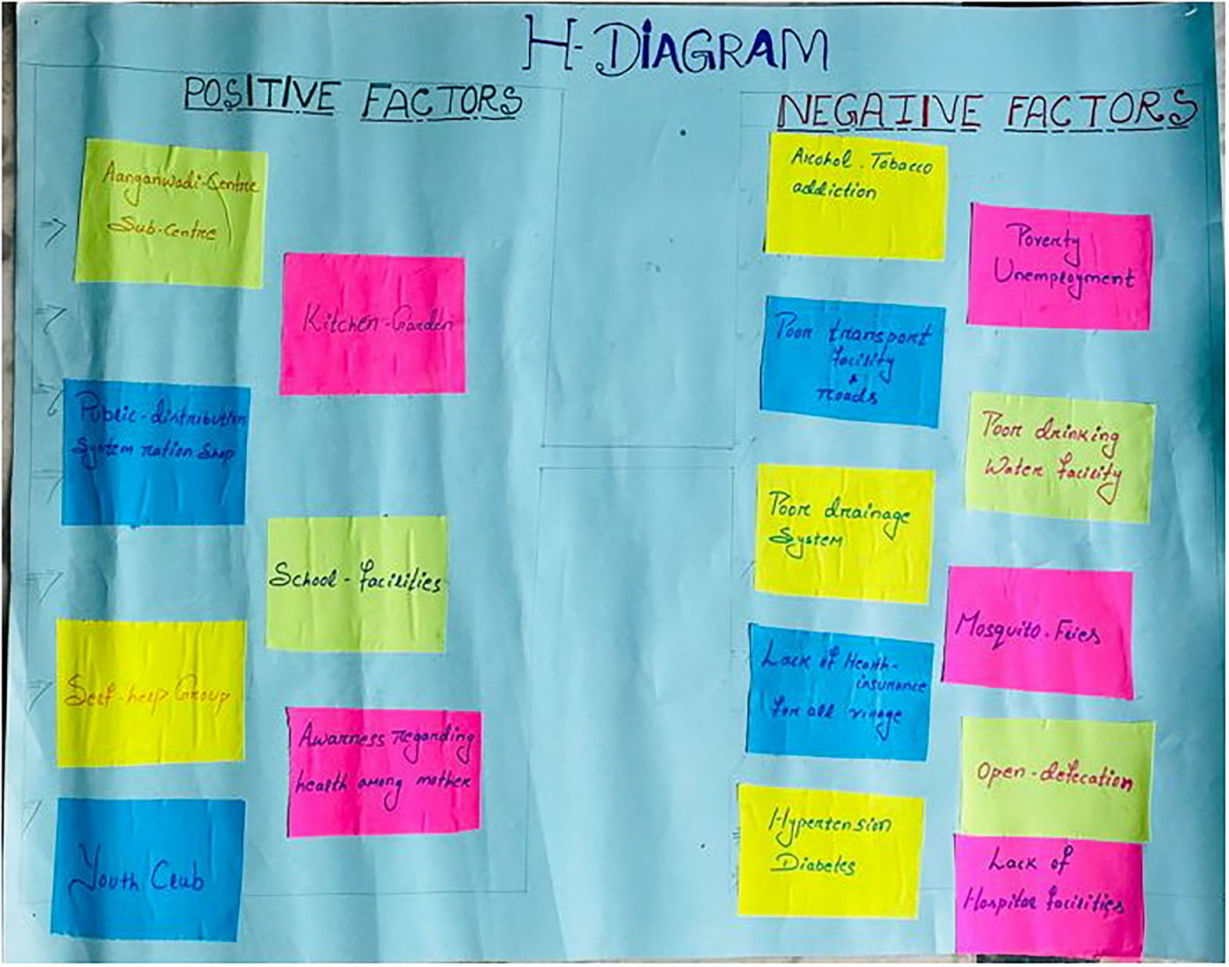
H-diagram.

**Figure 4:**
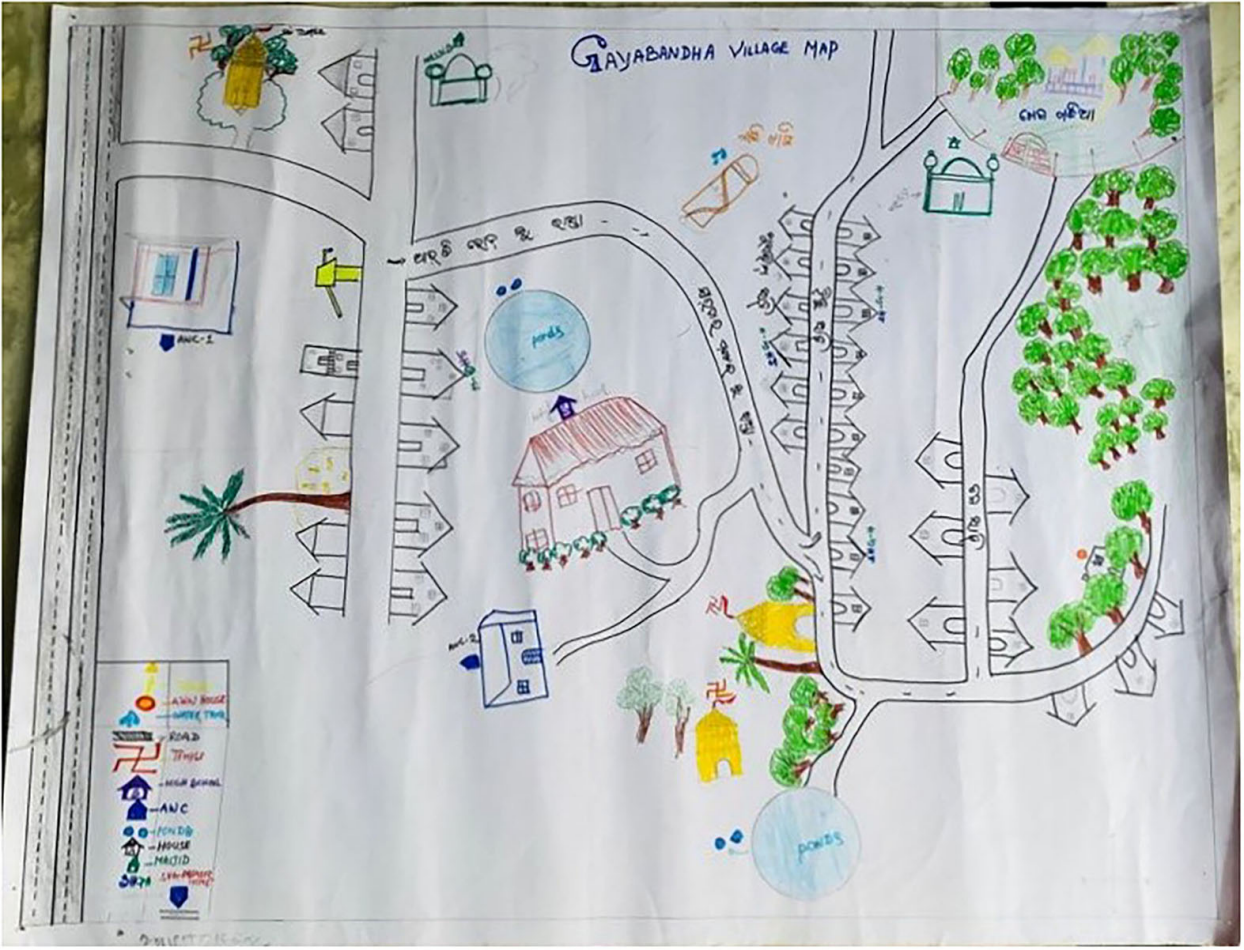
Resource map of a village.

**Figure 5:**
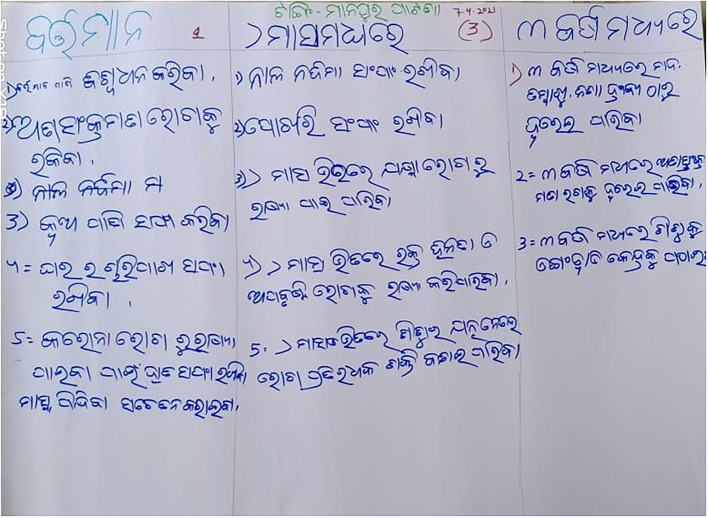
NSL Chart prepared by a team.

Each PLA exercise was facilitated by one member (medical social worker). of the research team trained in conducting participatory research. Once the invited participants had gathered at the meeting hall, the facilitators welcomed them and provided all the logistics required for PLA exercises. The role of the facilitator was to communicate the instructions and guide the team without interfering in the PLA process proper. Another member was assigned for the documentation and reporting of all the activities. Each team was given 3 h each to complete the exercises. The team also prepared detailed field notes including short non-formal interviews with the key participants. So, in total, 30 PLA exercises for the selected 10 villages have been included in the study (Figures 1–5).

#### Dissemination

After the completion of the PLA exercises, each team presented and summarized key findings from PLA session and invited feedback from other participants. It was a participant-driven discussion that was documented by our reporting team for further analysis.

### Data Analysis

We used the data from worksheets of PLA exercises and final dissemination in the form of open discussion among participants to construct conceptual categories which characterized major themes. A qualitative content analysis of the data was performed through deductive approach (26, 27). It was our objective that any generalized theoretical statements in the form of verbatims would be embedded in these thematic categories and subcategories. The Quirkos qualitative data analysis software was used to undertake coding, analysis, and data management for this study. The content analysis was done in the following ways. As the first step, the data were reviewed and transcripts were re-read to identify important codes under deductive approach. Then, the patterns and possible relationships between codes were identified and organized into categories and themes. The statements in the field notes were categorized and analyzed according to the frequency in general, frequency in each field note from different facilitators and controversial discussion of the statements from participants. Typical statements were marked and used for later citation through consensus among investigators. Analysis, interpretation, and conclusions were meticulously drawn describing the study context and the attributes of the study participants.

### Ethical Consideration

The written consent of the participants was taken and the study protocol was approved by Institutional Ethical Committee of AIIMS Bhubaneswar (IEC ref. no.: T/IM-NF/CM&FM21/53). In addition, permission for documentation of PLA sheets and discussions were obtained from all participants. Names of participants were excluded from the final datasets to ensure confidentiality and anonymity.

## Results

**Figure 6:**
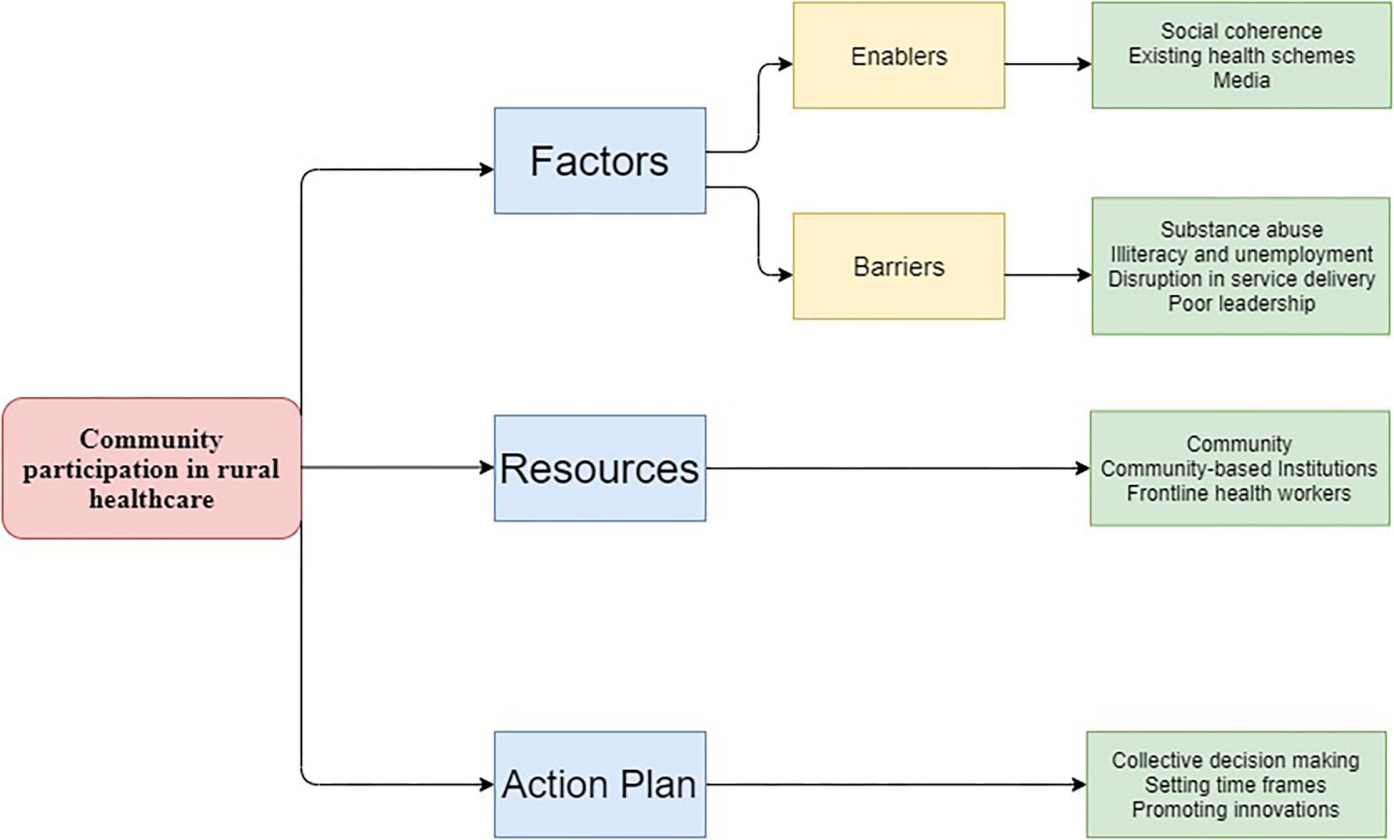
Community participation framework.

In this section, we present the key thematic findings from each of the three PLA techniques which were synthesized from facilitators' notes for each team as well as from our ethnographic observation. We identified three interrelated themes, i.e., factors, resources, and action plan, based on of stakeholders' experiences and perspective on community participation. The “Factor” theme was further divided into subthemes of barriers and enablers. The categories under each theme have been explained further in the result section. Based on the final analysis results, a framework for community participation with respect to rural health care was developed (Figure 6).

### The Factors

In this PLA exercise, community members across almost all villages spoke about the importance of identifying the enabler and barriers toward different aspects of health through the H-diagram.

### Enablers

Many participants identified the existing social coherence in their villages as one of the significant enablers of community participation.

“*In my village, there is a sense of belongingness to the society…Everybody feels to be a part of a larger family and tries to contribute in whatever way possible if needed by the village….” (PRI member)*

All the stakeholders reported that schemes such as Ujjwala Yojana (free LPG gas connection), Swachh Bharat Mission (free toilet), Public Distribution System (PDS), and so on have played a significant role in maintaining the health in their community. In addition to this, these schemes have also helped to build an enabling environment for community participation at the village level either directly or indirectly. Most of the participants echoed the fact that maternal and child health care have improved significantly in their villages due to community participation in public health programs and enhanced awareness within the female community.

“*When we think about delivery 8–10 years back, most of the families were preferring home deliveries and we had to counsel them to go to the hospital for institutional delivery…Now the government is providing so much financial and other types of support to pregnant mothers…. So, they are opting for delivery at hospitals. Even the number of mothers participating in VHNDs has also increased.” (ASHA)*

Most of the participants felt that the COVID-19 pandemic has made villagers more responsible toward disease prevention within their community. Many of them agreed that this pandemic has taught the villagers how health is a collective responsibility of everyone within their community. The local PRIs also feel more empowered due to the role played by them in managing this pandemic at the village level through community participation.

“*During this pandemic, the government empowered the PRIs in many ways to deal with it…As we are staying in villages, we are more motivated to keep it safe…Villagers also supported us in this fight against COVID except for few instances when we had to take the help of law.” (PRI member)*

Many participants emphasized the role of media and the internet in improving community participation in their villages as they have increased the availability of information as well as the speed of communication.

“*Now-a-days health-related information are available in news media and the internet. People are getting the right information at the right time which is motivating them to take part in various community-based health-related activities” (GKS member)*

Some of the stakeholders were less vocal about the enablers of community participation in their community as they were more concerned about the barriers. On the other hand, the participants also expressed a need for further improvement in some of the enabling factors of community participation highlighted by them.

### Barriers

Social challenges such as illiteracy, migration, unemployment, and the lack of awareness regarding health were described as the most important barriers toward establishing health as a shared responsibility among the rural population.

“*Villagers are so much entangled in the day-to-day issues that health becomes the least priority for them. Many villagers who are without regular employment are busy in their daily struggle for income.” (PRI member)*

Almost all the participants identified alcohol and other substance abuse as a significant challenge from a public health perspective in their community. They also highlighted that alcohol abuse leads to an increased incidence of other social evils such as domestic violence, sexual abuse, and so on. Problems such as substance abuse and local politics have eroded the social cohesion existing in villages. This in turn is hampering the environment for social mobilization as well as sustained collaboration required for community participation.

“*As the government is providing free ration to poor villagers, they are using their income in buying alcohol…It's not like they are unaware of ill effects of alcohol…but initially, they start it due to peer pressure and later they get addicted...So many people are getting admitted in hospital due to liver problems...” (SHG member)*

“*No major community awareness event has ever been implemented in our village related to prevention of alcohol abuse” (ASHA)*

As per some participants, disruption in service delivery has also affected the enthusiasm toward community participation among the village population. Dissatisfaction among the community regarding the quality of health care affects the motivation of villagers toward community participation. Some of the participants also highlighted that many from the rural population are depending on private health care for health services making them less enthusiastic toward community participation.

“*Previously we used to have more adolescents girls coming to VHNDs as we were distributing sanitary pads at affordable costs…But since few months we are not being able to provide them…this has affected the footfall of girls in VHNDs…” (SHG member)*

Among other barriers highlighted by the participants, the lack of leadership quality among PRIs and failure of government in solving some of the pre-existing problems related to basic health needs of villages have affected the involvement of villagers in community-based health events.

### The Resources

All the participant groups mapped the resources playing a significant role in maintaining health in their community through a resource mapping exercise. Only three out of 10 villages had subcenter in their village and none of the villages had a primary health center. Although most of the villages had primary health centers within manageable distance, poor road connectivity was one of the important issues highlighted by the teams. Few villages were having community centers but were seldom utilized for health-related events.

“*For most of the common ailments, our villagers go to the nearest Primary health centers… Due to poor road, it almost takes 1 h to reach the primary health center…On the other hand for community-based events related to health, we either use schools or Anganwadi ….” (PRI member)*

The most important resource for community participation in villages was the ASHAs and AWWs as agreed by most of the stakeholders, especially the PRI members and SHG members.

“*In our villages, people approach ASHAs first for any health issues as they are easily approachable and they are working very hard. People trust ASHA and Aanganwadi workers because they always try to create awareness about health within the community by involving villagers, especially women” (GKS member)*

Most of the ASHAs and AWWs were staying in the same locality and were well engaged with the community assigned to them. In case of any community-based activity, ANM and ASHA take the lead role in community mobilization. AWW, members from PRIs and SHGs, support them in reaching to villagers and creating positive opinions regarding the event within the society. Few participants reported that now-a-days school teachers are getting involved in health-related events such as the Measles-Rubella campaign, mass drug administration, and so on in villages. However, primarily, they are involved in teaching and providing nutritious food to school children through a mid-day meal program.

“*School teachers come to our village for survey and some other health-related campaigns…On the other hand, our children get mid-day meals in schools but here the school is quite far from our village…The food quality in schools can also be improved” (GKS member)*

As per the participants, most of the villagers were having farming and daily labor as an occupation. But they were dependent on the public distribution system (PDS). for subsidized monthly food supply through ration cards. On the other hand, it was voiced by some members that food security is no replacement for nutrition security and kitchen gardens are trying to fill this gap to some extent. Some other resources such as drinking water supply and proper drainage were mapped by the team members but during discussion, and they expressed their dissatisfaction due to poor accessibility and maintenance. They also agreed upon the need for villagers to take the responsibility for maintaining a clean and hygienic village environment.

“*Our village had drainage system for last 3 years but most of the time it remains blocked due to poor maintenance... Some villagers use this as an excuse to stay away from community-based cleanliness drives that we plan occasionally. Villagers dispose of all the wastes into drainage and nobody cares for its cleaning…” (ASHA)*

Most of the stakeholders were putting the responsibility on government administration for equipping the villages with sufficient resources needed for a healthy community. Many participants agreed that community mobilization needs sustained efforts and not just resources as it has a significant role in primary health care, especially in resource-limited settings.

“*The fund that we receive from government are very little to solve the problems…Crowdfunding is not an option always as there is a chance of corruption...Villagers should also be more responsible toward utilizing services. For example…Despite getting free toilets built in their backyards, people are still going for open defecation…” (PRI member)*

Most of the participants were able to identify the resources and their utilities already available in their villages in improving community participation. They considered local level institutions such as AWC, subcenter, schools, and panchayat office as the essential resources promoting community participation in their villages. However, they were lacking in orientation toward a structured approach for community participation in solving local problems and utilizing available resources.

### The Action Plan

All the teams were asked to prepare an action plan for community participation in their village using now, sooner, later (NSL). chart as a part of the final exercise. The participants were realistic in preparing the action plan as most of them were well aware of their limitations. The action plan was based on priority issues identified by the teams and resources available to their village. The majority of the teams planned to restart and strengthen existing activities such as adolescent health days, monthly mother's meetings, cleaning drainage, and so on within 6 months. Few SHG members suggested planning awareness campaigns within their community against issues such as alcohol abuse, early marriage, open defecation, and so on. Some even suggested innovative approaches such as creating social media groups, peer support groups among elderly patients, and increasing the number of “Swasthya Kantha” (village health awareness walls). in their villages within the next few months. Participants also planned to contribute vegetables from kitchen gardens to AWC, so that their children can get nutritious food even at AWC.

“*Now-a-days every household has a mobile phone. Why can't we form what's app groups and send awareness messages through them…We can plan meetings and discussions through them!!!....” (GKS member)*

Under long-term plans, some villages proposed collaborating with local NGOs in implementing their annual action plans for their community. Crowdfunding was also discussed as an option to implement low-cost solutions through proper planning and transparency. GKS members from some teams suggested improving the coverage of beneficial health-related schemes such as Biju Swasthya Kalyan Yojana (Biju health insurance scheme). and other social benefit schemes to get more community support. The majority of the teams also pointed out that they would promote physical activity and adoption of a healthy lifestyle through community participation as a part of their action plan to tackle NCDs in their community. All the villages, especially those teams with SHG members stressed working toward improving menstrual health in their community by strengthening adolescent health services in their community.

“*Our girls need to adopt better menstrual hygiene practices… I have read somewhere that the local SHGs in some of our villages are making sanitary napkins by themselves… We will also plan to do that in our village…but we need some training and funding…let's see how it proceeds…” (SHG member)*

Although all the team members were very much excited in preparing an action plan for their villages, many of the interventions they identified needed infrastructural changes (e.g., building roads, hospitals, etc.). All of them agreed to highlight these needs in front of the administration more sensibly and coherently.

“*Although our plans include many actions in near future…we will work more toward creating a sense of collective responsibility within community members as maintaining a healthy environment in our villages is the priority now for us…” (GKS member)*

“*No doubt that without government support we cannot act on our plans…But to change our community, we need to change ourselves first…We need to change our behaviors…Schemes are there…but we will work on its implementation so that more people get benefitted…” (PRI member)*

Most of the participants agreed that there is already an existing system for community participation in every village. Many participants stressed the need for regular village meetings with health as a priority topic to maintain a sense of urgency among the general population. Most of the teams also echoed the fact that the PLA technique can be very much useful in planning for healthcare interventions at the village level.

## Discussion

For all the participants, this exercise was a novel experience as most of them were used to a reactive work culture rather than a proactive one. Our findings demonstrate that employing the PLA mode of engagement and participatory techniques can result in the meaningful association of stakeholders who came from diverse backgrounds but work toward achieving a common goal. Already, there is enough evidence existing with regard to the effectiveness of the participatory approach in bringing improvement in the health status of the community (28, 29). However, incorporating it into the routine work culture and sustaining it would be more fruitful if we are to achieve effective community empowerment.

This study reinforced the fact that frontline health workers such as ASHAs are the face of the health system within the rural community and would continue to remain the same in the future (30). Similarly, It was observed that self-help groups (SHGs). have emerged as an essential component of community participation in the healthcare delivery system due to a broad population coverage and significant social capital produced by their activities in rural areas (31). But the GKS and PRI members were much more focused on the unavailability of resources rather than utilization of the available ones. This may be associated with poor multisectoral convergence and lack of leadership quality needed for healthcare delivery at the village level (32).

Many of the participants acknowledged that major communicable diseases had been tackled successfully through community participation. This has been backed by various Indian studies done in the field of undernutrition and communicable diseases (33–35). However, all the participants took note of the emergence of newer health-related challenges such as non-communicable diseases (NCDs), the ongoing COVID-19 pandemic as a significant threat to the health of their community which is obvious due to the epidemiological transition that has been witnessed at the global level including India (36). On the other hand, it has been recognized that community participation will play an even bigger role in tackling these future challenges. But, the frontline health workers still lack clarity on the role of community participation in NCD control, probably due to poor supportive supervision and implementation issues (37).

This study highlighted many common challenges related to health faced by villages but the PLA session prompted the attendees to think on solutions with the minimal resources available to them through community participation. This may help in activating the social accountability system within their community when they start working on their action plans. Through this process, the participants acknowledged that their strategy for health education must evolve from creating health awareness to achieving health literacy. A study on the tactics and effects of integrated community case management (iCCM). program in Niger and Mozambique demonstrated that service uptake can be improved through community involvement with a significant emphasis on demand generation (38). It was also perceived by most of the participants that community participation must focus on preventive strategies in addition to curative ones. On the other hand, it was also observed in our study that there is a sense of pessimism among participants, which needs to be addressed through sustained dialogue with political as well as administrative stakeholders (39, 40).

Community participation is widely believed to be beneficial to the planning, implementation, and evaluation of health services (15). It has potential advantages and consequences for community empowerment, with regard to health care, as well as promotion of locally appropriate services to reflect community needs (41). One study from rural Mexico found that patients did not have access to complete advanced treatment prior to the launch of the Right to Health Care Initiative, a community participation activity (42). However, this study showed that many challenges to successful and sustainable community involvement still remain. Francesca and Nicholas in a similar study done in India reported how mechanisms for community participation have been hampered due to confusion between accountability and decentralization functions of grassroot level institutions (43). Committees such as VHNSC, GKS, and so on are the ultimate representative of community participation in health. But, the presence of these community-based mechanisms must be added by professionalism and teamwork attitude to bring about transformation in their functioning. Similarly, there must be clarity on the path to be followed by the stakeholders in empowering these institutions with adequate resources (44).

We conclude that the PLA activity helped augment the knowledge, capacity, and self-confidence of participants to enact village health plans through community participation in a rational and effective manner. Participants credited this transition to the intervention and to the participatory approach which involved group members in critical thinking through need assessment and active dialogue. Although community participatory approaches in health have been undermined for a large duration due to its resource-intensive and complex nature, they can be a game changer if utilized logically. Participatory learning activities can become a potent intervention for addressing health issues at the village level by helping the community in shifting away from passive to active community participation. There is a need for reinforcement of public health responses to empower local organizations at the village level to complement community-based efforts. The health system needs to focus on skill upgradation and capability reinvigoration among all stakeholders of rural health care to provide adequate leadership required for community participation. Most important of all is the development of the “spirit” of participation in the society, which needs a balanced approach involving people, policy and pragmatism.

## Data Availability Statement

The raw data supporting the conclusions of this article will be made available by the authors without undue reservation upon reasonable request.

## Ethics Statement

The studies involving human participants were reviewed and approved by Institutional Ethical Committee, All India Institute of Medical Sciences, Bhubaeswar. The patients/participants provided their written informed consent to participate in this study. Written informed consent was obtained from the individual(s) for the publication of any potentially identifiable images or data included in this article.

## Author Contributions

AM, SKP, and JN did the literature search, data acquisition, data analysis, and manuscript preparation. AM, AS, SPP, SKP, and JN contributed in manuscript editing, manuscript review, concepts, design, and definition of intellectual content. All authors contributed to the article and approved the submitted version.

## Conflict of Interest

The authors declare that the research was conducted in the absence of any commercial or financial relationships that could be construed as a potential conflict of interest.

## Publisher's Note

All claims expressed in this article are solely those of the authors and do not necessarily represent those of their affiliated organizations, or those of the publisher, the editors and the reviewers. Any product that may be evaluated in this article, or claim that may be made by its manufacturer, is not guaranteed or endorsed by the publisher.
